# Individual- and country-level correlates of female permanent contraception use in sub-Saharan Africa

**DOI:** 10.1371/journal.pone.0243316

**Published:** 2020-12-15

**Authors:** Babayemi O. Olakunde, Jennifer R. Pharr, Lung-Chang Chien, Rebecca D. Benfield, Francisco S. Sy

**Affiliations:** 1 Department of Environmental and Occupational Health, School of Public Health, University of Nevada, Las Vegas, NV, United States of America; 2 Department of Community Prevention and Care Services, National Agency for the Control of AIDS, Abuja, Nigeria; 3 Department of Epidemiology and Biostatistics, School of Public Health, University of Nevada, Las Vegas, NV, United States of America; 4 School of Nursing, University of Nevada, Las Vegas, NV, United States of America; Ohio University College of Health Sciences and Professions, UNITED STATES

## Abstract

**Background:**

Female permanent contraception is a cost-effective contraceptive method that can help clients with the desire to limit childbearing achieve their reproductive intention. However, despite its benefits, the use of FPC remains low in sub-Saharan Africa (SSA), and limited studies have examined the correlates of its uptake. In this study, we assessed the individual- and country-level factors associated with the use of FPC among married or in-union women using modern contraceptive methods to limit childbearing in SSA.

**Methods:**

This study was a secondary data analysis of individual- and country-level data obtained from the Demographic and Health Surveys (DHS) Program and three open data repositories. The study included 29,777 married or in-union women aged 15–49 years using modern contraceptive methods to limit childbearing from DHS conducted in 33 sub-Sahara African countries between 2010 and 2018. We performed descriptive statistics and fitted multilevel logistic regression models to determine the predisposing, enabling, and need factors associated with the use of FPC.

**Results:**

Approximately 13% of the women used FPC. About 20% of the variance in the odds of using FPC was attributable to between-country differences. In the full model, the significant individual-level factors associated with the use of FPC compared with other modern contraceptive methods were: age (odds ratio [OR] = 1.10; 95%CI = 1.08–1.12), living children (OR = 1.11, 95%CI = 1.04–1.16), high household wealth (OR = 1.39, 95%CI = 1.18–1.64), rural residence (OR = 0.83, 95% CI = 0.71–0.97), joint contraceptive decision with partner (OR = 1.68, 95% = 1.43–1.99), contraceptive decision by partner and others (OR = 2.46, 95% = 1.97–3.07), and the number of living children less than the ideal number of children (OR = 1.40, 95%CI = 1.21–1.62). The significantly associated country-level factors were births attended by skilled health providers (OR = 1.03, 95%CI = 1.00–1.05) and density of medical doctors (OR = 1.37, 95%CI = 1.01–1.85).

**Conclusions:**

Our results suggest that both individual- and country-level factors affect uptake of FPC in SSA. Increasing geographic, economic, and psychosocial access to FPC may improve its uptake in SSA.

## Background

Despite the increasing proportion of women with demand to limit childbearing in sub-Saharan Africa (SSA) [1, 2], the uptake of female permanent contraception (FPC) which offers a cost-effective, convenient, and safe method to limit births, remains low [3, 4]. The majority of these women do not use contraceptives or rely on reversible methods that are not as effective as FPC [4], thereby increasing their risk of having unintended pregnancies [5, 6]. Estimates indicate that approximately one in three pregnancies in SSA is unintended [7]. Unintended pregnancies are responsible for nearly 30% of maternal deaths and 26% of newborn deaths in SSA [7]. They also have negative social and economic implications [8]. Higher proportion of unintended pregnancies have been found among women who have a desire to limit compared to space childbirths [9].

Globally, FPC is the most widely used contraceptive method [10, 11], representing 24% of the method mix among women using contraceptives [11]. It is the most commonly used method in regions like Central and Southern Asia, and Latin America and the Caribbean [10]. Compared to other regions, SSA has the lowest uptake of FPC in the world, where it accounts for less than 3.9% of the method mix [11].

While women face multilevel barriers in accessing modern contraceptive methods in SSA, how these factors affect uptake of FPC have not been comprehensively examined [12]. A number of quantitative studies that have assessed the use of FPC in SSA conflated FPC with other long-acting reversible methods. Thus, findings reported in these available studies are not specific to FPC. Furthermore, a majority of the studies considered only individual-level factors, leaving out contextual factors which may also affect the uptake of FPC [13, 14].

A better understanding of the factors that influence the uptake of FPC is needed to improve its voluntary and informed utilization among clients who want to limit childbearing in SSA [15]. Accordingly, this study aimed to add to the sparse body of evidence on the use of FPC in SSA by providing insight into its correlates. The objective was to assess the individual- and country-level factors associated with the use of FPC among married or in-union women using modern contraceptive methods to limit childbearing in SSA.

## Methods

### Conceptual framework

**Fig 1:**
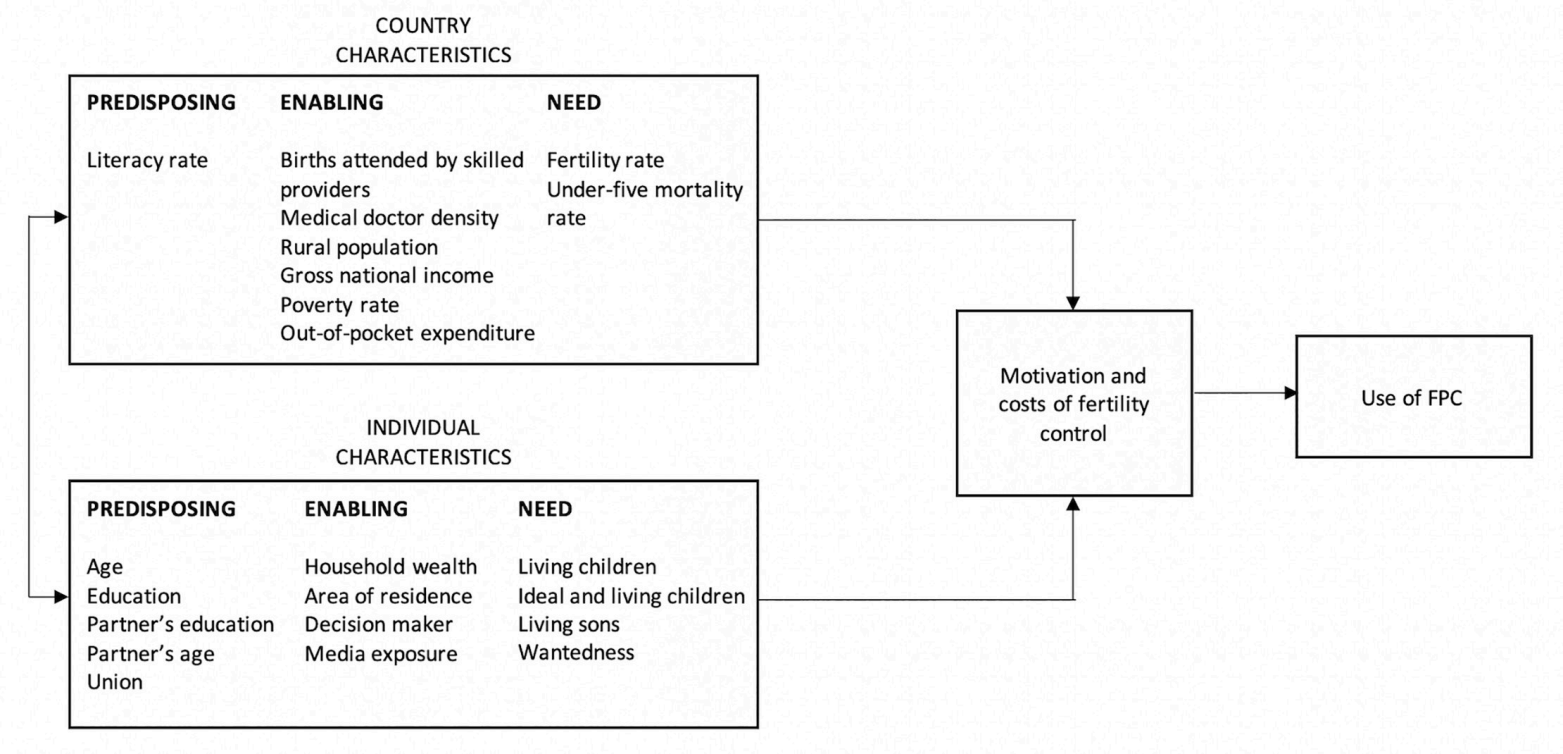
Conceptual framework.

This study drew upon the supply-demand framework for the determinants of fertility and contraceptive use [16, 17] and the behavioral model of health services use [18, 19] to assess the correlates of the use of FPC among married and in-union women in SSA. According to the supply-demand framework for the determinants of fertility and contraceptive use, the motivation or incentive for fertility regulation is influenced by both the demand for and supply of children [16, 17]. If supply is lower than demand, there would not be a desire to limit fertility and vice versa [16, 17]. However, the framework holds that the use of fertility control when there is excess supply would depend on the costs (subjective and objective) of obtaining and using a contraceptive method relative to the motivation to limit fertility [16, 17]. The behavioral model of health services use (also known as the Andersen model) posits that “people's use of health services is a function of their predisposition to use services, factors which enable or impede use, and their need for care” [19, p1]. Recognizing the significance of community, structure, and process of service delivery, Andersen later added contextual characteristics as important determinants of health behaviors in the revised version of the model [20]. Andersen noted that, “understanding of utilization of health services can be best achieved by focusing on contextual and individual determinants” [20, p652]. The components of contextual characteristics are similar to individual characteristics (predisposing, enabling, and need factors) [20], but they are measured at an aggregate level (e.g., community or country). Contextual factors can affect individual factors, which will in turn influence health behaviors and outcomes, or they can directly influence health behaviors and outcomes. The addition of contextual characteristics to the model has been described as one of its strengths, and makes it appropriate for multilevel models to assess the determinants of health care utilization [21]. The predisposing, enabling, and need variables considered in this study are factors that have been empirically found to influence the use of contraception or are theoretically plausible (Fig 1).

### Study design and sample

This study was a secondary data analysis of data obtained from the Demographic and Health Surveys (DHS) Program and three open data repositories. The study included 29,777 married or in-union women of reproductive age (15–49 years) who were using modern contraceptive methods (i.e., condom, diaphragm, emergency contraception, female condom, female permanent contraception, foam and jelly, implants, intrauterine device, injections, lactational amenorrhea, male permanent contraception, pill, or standard day method) to limit childbearing.

### Data sources

The Individual-level data were obtained from the DHS Program (https://dhsprogram.com/). DHS are nationally-representative household surveys that gather data on a number of health-related topics. The DHS program utilizes standardized methodologies and procedures, making the surveys comparable across different countries. The DHS program adopts a stratified two-stage probabilistic sampling design. More details about the survey procedure can be found elsewhere [22]. SSA countries with a standard DHS conducted in the last 10 years (between 2010 and 2019) were considered for inclusion in this study. Based on these inclusion criteria, 33 countries were included, with the years of the surveys ranging from 2010 to 2018 (Table 1). The country-level data were publicly available data obtained from three open data repositories: the World Bank (https://www.worldbank.org/), World Health Organization (WHO) (https://www.who.int/), and United Nations Educational, Scientific and Cultural Organization (UNESCO) (https://en.unesco.org/). We used the most recent available data corresponding or closest to the DHS survey year for each of the countries.

**Table 1:** Summary of included DHS.

S/N	Country	Survey Year	Married or in-union women using modern contraceptive methods to limit childbearing
1	Angola	2015–16	243
2	Benin	2017–18	532
3	Burkina Faso	2010	694
4	Burundi	2016–17	1073
5	Cameroun	2011	498
6	Chad	2014–15	131
7	Comoros	2012	129
8	Congo	2011–12	211
9	Cote d’Ivoire	2011–12	209
10	Democratic Republic of Congo	2013–14	308
11	Ethiopia	2016	1073
12	Gabon	2012	196
13	Gambia	2013	158
14	Ghana	2014	516
15	Guinea	2018	173
16	Kenya	2014	2418
17	Lesotho	2014	1332
18	Liberia	2013	401
19	Malawi	2015–16	5010
20	Mali	2018	335
21	Mozambique	2011	581
22	Namibia	2013	1142
23	Niger	2012	162
24	Nigeria	2018	1471
25	Rwanda	2014–15	1656
26	Senegal	2017	794
27	Sierra Leone	2013	592
28	South Africa	2016	1058
29	Tanzania	2015–16	911
30	Togo	2013–14	471
31	Ugandan	2016	1749
32	Zambia	2013–14	1686
33	Zimbabwe	2015	1864

### Measures

#### Dependent and explanatory variables

A binary dependent variable was created from married or in-union women (15–49 years) using modern contraceptive methods to limit childbearing. Married or in-union women using FPC were coded ‘1’, while those using other modern contraceptive methods were coded ‘0’. The explanatory variables were categorized into two levels: individual and country (Fig 1). Under each level, the variables were grouped into predisposing, enabling, and need. See S1 Table for operational definitions of the variables.

### Data analysis

Crude and weighted descriptive statistics were performed to summarize the data. For the weighted percentages, we de-normalized the weights for each of the countries [23], using the population of women of reproductive age (15–49 years) (https://population.un.org/wpp/Download/Standard/Population/) corresponding to the year of survey. We performed a 2-level multilevel logistic regression analysis, with individual-level factors at level one and country-level factors at level two. Four random intercept models were fitted as follows: Model 1 (an empty model with no variable); Model 2: (individual-level variables with survey year as a control variable); Model 3 (country-level variables with survey year as a control variable); and Model 4 (individual- and country-level variables with survey year as a control variable). Multicollinearity among the explanatory variables was examined using the variance inflation factor, with a value exceeding 10 used as the cut-off [24]. Multicollinearity was not detected among explanatory variables. Fixed effects (measures of association) were reported as odds ratios (ORs), with their 95% confidence intervals (CIs). Random effects (measures of variation) were measured by the intra-cluster (i.e. intra-country) correlation coefficient (ICC), proportional change in variance (PCV), and median odds ratio (MOR) [25–28]. Responses such as ‘don’t know’ and nonnumeric responses to questions that required numeric answers were treated as missing data. The analysis was conducted using listwise deletion (complete-case analysis). P-value of less than 0.05 was considered statistically significant. The data analysis was performed using Stata Statistical Software: Release 15, College Station, TX, StataCorp LLC.

### Ethical consideration

This study was a secondary analysis of publicly available data. All the data used were fully anonymized. The DHS program obtains ethical clearance from appropriate National Ethics Committees in the respective countries before conducting the surveys. Access and permission to use the datasets was granted by ICF. This study was deemed exempt by the University of Nevada, Las Vegas Institutional Review Board.

## Results

### Descriptive statistics

The individual-level characteristics are summarized in Table 2. The mean age was 36.4 years. About 38% of the respondents had at least secondary education. Approximately 51% of the respondents were from rich households and 57% resided in a rural area. Of the 29,777 married or in-union women using modern contraceptive methods to limit childbearing, 12.7% used FPC (Table 2). The use of FPC varied by individual-level characteristics. It was higher than 12.7% among women who: were ≥40 years, had at least a primary education, were from rich households, were urban dwellers, had ≥ 3 living children, whose living children were less than ideal their number of children, had ≥ 3 living sons, were exposed to family planning messages, made joint contraceptive decision with husband/partner, whose husband/partner or others made contraceptive decision, whose husband/partner was ≥40 years, whose husband/partner had at least primary education, were in a polygynous union, and wanted no more children at last birth. Table 3 presents the summary of the country-level characteristics. The mean total fertility rate was 4.9 while the mean percentage of births attended by skilled health providers 66.2%. The mean density of medical doctors was 1.4 per 10,000 population.

**Table 2:** Descriptive statistics of individual-level characteristics.

Characteristics	Total		FPC		Other modern methods	
	N (%)	Weighted %	N (%)	Weighted %	N (%)	Weighted %
All	29777 (100)	100	4022 (13.5)	12.7	25755 (86.5)	87.3
Age (N = 29777); Mean (SD)^a^	36.3 (6.8)	36.4 (6.8)	40.3 (5.6)	40.6 (5.5)	35.8 (6.8)	35.8 (6.7)
Education (N = 29777)						
None	6539 (22.0)	24.6	829 (12.7)	9.8	5710 (87.3)	90.2
Primary	13419 (45.1)	37.0	2069 (11.4)	14.0	11350 (84.6)	86.0
Secondary or higher	9818 (33.0)	38.3	1124 (13.5)	13.2	8694 (88.6)	86.8
Household wealth (N = 29777)						
Poor	9321 (31.3)	28.8	1083 (11.6)	11.1	8238 (88.4)	88.9
Middle	5926 (19.9)	20.0	774 (13.1)	11.7	5152 (86.9)	88.3
Rich	14530 (48.8)	51.2	2165 (14.9)	13.9	12365 (85.1)	86.1
Area of residence (N = 29777)						
Urban	11471 (38.5)	42.7	1386 (12.1)	12.9	10085 (87.9)	87.1
Rural	18306 (61.5)	57.3	2636 (14.4)	12.5	15670 (85.6)	87.5
Living children (N = 29777); Mean (SD)	4.5 (1.9)	4.4 (1.9)	4.9 (2.0)	4.7 (2.1)	4.4 (1.9)	4.4 (1.9)
Ideal and living children (N = 28870)						
Living equal or greater than ideal	19748 (68.4)	66.9	2517 (12.7)	12.2	17231 (87.3)	87.8
Living less than ideal	9122 (31.6)	33.1	1321 (14.5)	13.7	7801 (85.5)	86.3
Number of living sons (N = 29777); Mean (SD)	2.3 (1.4)	2.2 (1.4)	2.5 (1.5)	2.4 (1.5)	2.2 (1.4)	2.2 (1.4)
Media exposure (N = 29771)						
Yes	16363 (55.0)	53.9	2311 (14.1)	13.6	14052 (85.9)	86.4
No	13408 (45.0)	46.1	1711 (12.8)	11.5	11697 (87.2)	88.5
Decision maker (N = 29651)						
Joint decision	19139 (64.5)	62.5	2717 (14.2)	13.3	16422 (85.8)	86.7
Mainly respondent	7570 (25.5)	28.2	688 (9.1)	9.0	6882 (90.9)	91.0
Mainly husband/partner or Others	2942 (9.9)	9.2	590 (20.1)	18.9	2352 (79.9)	81.1
Husband/Partner’s age (N = 29653); Mean (SD)	43.2 (9.5)	43.5 (9.4)	46.8 (8.5)	47.1 (8.7)	42.6 (9.5)	43.0 (9.4)
Husband/Partner’s education (N = 29212)						
None	5023 (17.2)	17.0	520 (10.4)	8.2	4503 (89.6)	91.8
Primary	11655 (39.9)	36.5	1908 (16.4)	14.5	9747 (83.6)	85.5
Secondary or higher	12534 (42.9)	36.5	1539 (12.3)	12.9	10995 (87.7)	87.1
Union (N = 29047)						
Monogynous	24074 (82.9)	84.9	3279 (13.6)	12.7	20795 (86.4)	87.3
Polygynous	4973 (17.1)	15.1	655 (13.2)	12.9	4319 (86.8)	87.1
Wantedness (N = 18798)						
Wanted then	10712 (57.0)	58.9	738 (6.9)	6.9	9974 (93.1)	93.1
Wanted later	3975 (21.1)	19.2	250 (6.3)	6.1	3725 (93.7)	93.9
Wanted no more	4111 (21.9)	21.9	454 (11.0)	10.2	3657 (89.0)	89.8

^a^ SD = Standard deviation.

**Table 3:** Descriptive statistics of country-level characteristics.

Characteristics	Mean (SD)^a^	Range
Poverty rate (%)	40.5 (19.0)	3.4–76.6
Literacy rate (%)	53.6 (23.0)	14–88
Births attended by skilled health providers (%)	66.2 (19.3)	20–97
Density of medical doctors (per 10,000 population)	1.4 (1.5)	0.2–8.0
Gross national income (USD)	1617 (1919)	270–9080
Rural population (%)	60.8 (17.2)	13–88
Total fertility rate (per woman)	4.9 (1.0)	2.5–7.4
Out-of-pocket expenditure (%)	37.2 (19.9)	8–78
Under-five mortality rate (per 1,000 live births)	79.8 (27.4)	36.6–136.7

^a^ SD = Standard deviation.

### Multilevel logistic regression analyses

#### Measures of variations (random effects)

From the fixed intercept of the empty model, the odds of using FPC in a typical country was 0.09 (not shown). However, the odds of using FPC varied considerably across the countries. As shown in Model 1 (empty model), there was a significant variation in the odds of using FPC across the 33 countries (*σ*^2^ = 0.82, 95% CI = 0.49–1.36) (Table 4). The ICC indicated that approximately 20% of the variance in the odds of using FPC to limit childbearing was accounted for by the countries in the study, while 80% of the variance was accounted for by the individual or other unknown factors. The MOR of 2.36 in the empty model also indicated considerable heterogeneity between the countries (Table 4). If a woman moved to another country with a higher probability of FPC use, their odds of using FPC would (in median) increase 2.36 times. In the full model (Model 4), after adjusting for the individual- and country-level factors and survey year, the variation in the odds of using FPC across the countries remained significant (*σ*^2^ = 0.42, 95% CI = 0.23–0.76). The ICC decreased to 11% and there was a reduction in MOR to 1.85 (Table 4). The full model showed that 49% of the variance in the odds of FPC in the empty model was attributable to the individual- and country-level factors considered in this study (Table 4).

**Table 4:** Multilevel logistic regression models of factors associated with the use of FPC compared with other modern contraceptive methods among married or in-union with desire to limit childbearing.

Variables	Model 1 OR^b^ (95% CI^c^)	Model 2^a^ OR (95% CI)	Model 3^a^ OR (95% CI)	Model 4^a^ OR (95% CI)
**Fixed effects**				
***Individual-level factors***				
Age (P)^d^		1.10 (1.08–1.12)***		1.10 (1.08–1.12)***
Education (P)				
None		Reference		Reference
Primary		1.00 (0.84–1.20)		0.99 (0.83–1.19)
Secondary or higher		1.03 (0.81–1.30)		1.00 (0.79–1.27)
Husband/partner’s age (P)		1.01 (1.00–1.01)		1.01 (1.00–1.01)
Husband/Partner’s education (P)				
None		Reference		Reference
Primary		1.12 (0.91–1.38)		1.11 (0.90–1.37)
Secondary or higher		1.13 (0.91–1.45)		1.14 (0.90–1.44)
Union (P)				
Monogynous		Reference		Reference
Polygynous		0.93 (0.78–1.10)		0.93 (0.79–1.11)
Household wealth (E)^e^				
Poor		Reference		Reference
Middle		0.97 (0.81–1.12)		0.97 (0.82–1.16)
Rich		1.38 (1.17–1.63)***		1.39 (1.18–1.64)***
Area of residence (E)				
Urban		Reference		Reference
Rural		0.83 (0.71–0.97)*		0.83 (0.71–0.97)*
Decision maker (E)				
Mainly respondent		Reference		Reference
Joint decision		1.68 (1.43–1.99)***		1.68 (1.43–1.99)***
Mainly husband/partner or others		2.45 (1.96–3.07)***		2.46 (1.97–3.07)***
Media exposure (E)				
No		Reference		Reference
Yes		1.00 (0.88–1.14)		1.00 (0.88–1.14)
Living children (N)^f^		1.10 (1.04–1.16)**		1.11 (1.04–1.16)***
Number of sons (N)		1.03 (0.97–1.08)		1.03 (0.97–1.08)
Ideal and living children (N)				
Living equal or greater than ideal		Reference		Reference
Living less than ideal		1.39 (1.20–1.61)***		1.40 (1.21–1.62)***
Wantedness (N)				
Wanted then		Reference		Reference
Wanted later		0.97 (0.82–1.15)		0.97 (0.82–1.14)
Wanted no more		1.13 (0.98–1.31)		1.12 (0.97–1.30)
***Country-level factors***				
Literacy rate (P)			1.01 (0.99–1.03)	1.01 (0.99–1.04)
Poverty rate (E)			0.98 (0.96–1.00)	0.98 (0.96–1.11)
Births attended by skilled health providers (E)			1.03 (1.01–1.05)*	1.03 (1.00–1.05)*
Density of medical doctors (E)			1.30 (0.98–1.72)	1.37 (1.01–1.85)*
Gross national income (E)			1.00 (1.00–1.00)	1.00 (0.96–1.01)
Out-of-pocket expenditure (E)			0.99 (0.97–1.01)	0.99 (0.96–1.01)
Rural population (E)			1.01 (0.98–1.04)	1.01 (0.98–1.04)
Total fertility rate (N)			1.71 (1.11–2.66)*	1.31 (0.81–2.14)
Under-five mortality rate (N)			1.01 (0.99–1.03)	1.00 (0.98–1.03)
**Random effects**				
***Country level***				
Variance (SE)^g^	0.82 (0.21)***	0.67 (0.21)***	0.43 (0.12)***	0.42 (0.19)***
95%CI	(0.49–1.36)	(0.38–1.22)	(0.25–0.73)	(0.23–0.76)
PCV (%)^h^	Reference	18.29	47.56	48.78
ICC (%)^i^	19.86 (12.92–29.27)	16.89 (10.03–27.03)	11.50 (7.11–18.06)	11.25 (6.51–18.78)
MOR^j^	2.36	2.17	1.86	1.85

^a^ Models 2–4 adjusted for survey year.

^b^ OR = Odds ratio.

^c^ Confidence interval.

^d^ P = Predisposing; ^e^ E = Enabling; ^f^ N = Need.

^g^ SE = Standard error. Standard error was used to calculate one-tail p-value for the variance.

^h^ PCV = Proportional change in variance; ^i^ ICC = Intra-country correlation coefficient. ^j^ MOR = Median odds ratio.

***p <0.001

**p <0.01

*p<0.05.

#### Measures of associations (fixed effects)

The fixed effects are shown in Table 4. In Model 2, age, living children, household wealth, area of residence, decision maker, and ideal versus living children were statistically significant. Births attended by skilled health providers and total fertility rate were the only significant factors in Model 3. In the full model (Model 4) that included all the individual- and country-level factors, after adjusting for survey year, all the individual-level factors that were significant in Model 2 (age, living children, household wealth, area of residence, decision maker, and ideal versus living children) remained statistically significant. While for country-level factors, births attended by skilled health providers remained significant as in Model 3, but total fertility rate was no longer statistically significant. However, density of medical doctors which was not significant in Model 3 became significant in the full model. For every one unit increase in age, the odds of using FPC increased by 1.10 (95% CI = 1.08–1.12). Also, for every one unit increase in the number of living children, the odds of using FPC increased by 1.11 (95% CI = 1.04–1.16). Compared to women from poor households, those from rich households had higher odds of using FPC (OR = 1.39, 95% CI = 1.18–1.64). Women residing in rural areas had lower odds of using FPC compared to those who resided in urban areas (OR = 0.89, 95% CI = 0.71–0.97). Women whose partners or others made contraceptive decisions (OR = 2.46, 95% CI = 1.97–3.07) or who made joint contraceptive decisions with their partners (OR = 1.68, 95% CI = 1.43–1.99) had higher odds of using FPC compared to women who made the decision by themselves. The odds of using FPC was significantly higher among women whose number of living children was less than their ideal number of children (OR = 1.40, 95% CI = 1.21–1.62). For every one unit increase in births attended by skilled health providers, the odd of using FPC increased by 1.03 (95% CI = 1.00–1.05). Similarly, for every one unit increase in the density of medical doctors, the odd of using FPC increased by 1.37 (95% CI = 1.01–1.85).

## Discussion

Using data from DHS program and three open data repositories, we investigated the individual- and country-level predisposing, enabling, and need factors associated with FPC use among married or in-union women aged 15–49 years who used modern contraceptive methods to limit childbearing in 33 countries in SSA. Approximately 13% of the women used FPC to limit childbearing. Individual-level predisposing, enabling, and needs factors and country-level enabling factors were found to be associated with the use of FPC among married or in-union women who were using modern contraceptive methods to limit childbearing.

FPC is a cost-effective contraceptive method that can help clients with the desire to limit childbearing achieve their reproductive intention. However, our study reaffirms that the majority of women using modern contraceptive to limit childbearing in SSA do not use FPC [4]. Although the classification of modern contraceptive methods may vary across studies [29], our finding was similar to a previous study which showed that from 2000–2005, FPC accounted for 11% of the modern contraceptive method mix among married women in SSA [30]. Our result is also comparable to the findings from 17 European countries. Using data from the Generations and Gender Survey and DHS from 2004–2011, Dereuddre and colleagues reported that FPC accounted for 10% of the modern contraceptive method mix among women of reproductive age who had a male partner and no childbearing intention in Western, Central, and Eastern Europe [31]. Indeed, the prevalence of FPC in some African countries matches or exceeds the prevalence in some European countries [11]. Our finding on the use of FPC in SSA is, however, low compared to Latin American/Caribbean and Asian countries where FPC accounted for 50% and 47% of the modern contraceptive method mix among married women, respectively from 2000–2005 [30].

There is a dearth of studies on correlates of FPC conducted at regional levels for comparison. However, some of the findings in this study are consistent with similar studies at country levels that have reported positive association between age [14, 32–39], number of living children [35], economic status [37, 40], and urban residence [14, 37] and the use of FPC. Being a permanent contraceptive method, FPC may be less appealing to younger women whose fertility preference may change overtime [41]. Provider bias towards younger women may also account for their lower odds of using FPC to limit childbearing [42]. Owing to the high demand for children in SSA, women with fewer living children may not been willing to permanently limit birth, as the need for children or more children may arise following life circumstances such as the death of a child or remarriage. Policies and laws may also require women to have a specified number of living children before they can have FPC [43]. In settings where FPC requires a high out-of-pocket payment, clients with low income may not be able to afford it. Even in countries where FPC services are free, clients may still incur high out-of-pocket expenditure [44]. In developing countries, FPC services are less likely to be available and accessible in rural areas [45]. However, in developed countries like the U.S., rural dwellers have been found to have higher odds of using FPC [33, 36]. While the reasons for the higher prevalence in rural areas of developed countries are not clear, Lunde et al. opined that it might be due to less access to reversible methods [36].

Contrary to our expectation, the results showed that women who had a lower number of living children than their ideal number of children were more likely to use FPC. This observation cannot be easily explained, and it needs to be further explored in future studies. Nonetheless, “unrealized fertility” is common in SSA [46, 47], and factors such as socioeconomic constraints, competing alternatives, or health issues may make clients opt for permanent contraception despite not having achieved their desired number of children [47].

These results also established the influential role male partners and others, such as peers, relatives, or healthcare providers, play in decision making with regard to contraceptive use and choice in Africa [12, 48–51]. Power imbalances as a result of traditional socio-cultural norms, economic differences, and age disparities allow male partners to influence reproductive health decision making in patriarchal societies that exist in many Africa countries [52, 53]. Given the permanent nature of the procedure, married or in-union women may be reluctant to make the decision alone to avoid consequences such as intimate partner violence, separation, divorce, and extramarital affairs [54–56].

Although the cadre of personnel that constitute skilled birth attendants differ by country [57], a positive relationship between births attended by skilled birth attendants and contraceptive use has been previously reported in studies conducted at the individual level [58, 59]. As a contextual enabling factor in this study, this may reflect access to FPC services in the countries we assessed. Also, with the positive correlation between antenatal care visits (particularly at least four visits) and births attended by skilled birth attendants in SSA [60–62], it is possible that in countries where a high proportion of births are attended by skilled birth attendants, women may have received information about FPC as an effective contraceptive option to limit childbearing during antenatal care, thereby increasing their preference for it [63]. In Brazil, receiving antenatal care was found to be positively associated with the use of FPC [64]. The higher of odds of using FPC in countries with more doctors also illustrates the importance of access to the method, which is largely obtained through surgical procedures [65]. Although task sharing to non-doctors such as clinical officers and other mid-level providers as recommended by the World Health Organization (WHO) [66] is increasing [67–69], there are still restrictions on health care cadres that can perform FPC in many countries with shortages of doctors [3].

Our study is not without limitations. Considering the cross-sectional nature of the survey, causality cannot be inferred from the findings. There is also possibility of underreporting of FPC among some of the women due to its sensitivity [70]. The surveys included in the study were conducted at different time points. However, this limitation was minimized by controlling for the year of survey in the regression models. Also, for some of the country-level variables, the most recent available data did not correspond with the DHS survey year. Although, initially included as some of the important individual-level factors to be explored, religion and insurance coverage were eventually excluded from the analysis because they were not reported in all the countries. These findings are also not generalizable to all women. The study was restricted to married or in-union women because of the assumption that they are at increased risk of pregnancy and may be more interested in limiting childbearing compared to unmarried women. Cultural sensitivity in some African settings may also result in reporting bias on contraceptive use among unmarried women which was another reason for their exclusion [71].

## Conclusions

Our results show that individual- and country- level factors are associated with the uptake of FPC in SSA. These factors suggest that increasing geographic, economic, and psychosocial access to FPC may improve its use among clients who desire to limit childbearing in SSA. Interventions such as mobile outreach to rural areas, reduced cost of FPC services, task shifting to lower cadre providers, male partner engagement, and joint family planning counseling may help in addressing some of these factors. Individual-level and other contextual factors specific to each country need to be examined. Qualitative studies are needed to understand the decision-making process among women and their male partners who choose to use FPC.

## Supporting information

S1 Table Description of explanatory variables. (DOCX)
