# Supplementary material for: Individual- and country-level correlates of female permanent contraception use in sub-Saharan Africa
Source: PLoS One. 2020 Dec 15;15(12):e0243316. doi: 10.1371/journal.pone.0243316 (PMC7737965; doi:10.1371/journal.pone.0243316)
Supplement: S1 Table — (DOCX) [file pone.0243316.s001.docx]

**S1 Table. Description of explanatory variables**

| Variable | Operational definition | Type of variable | Categories and coding |
| --- | --- | --- | --- |
| **Individual level** | | | |
| ***Predisposing*** | | | |
| Age | Age of the respondent in years at interview | Continuous |  |
| Education | Highest educational level attained by the respondent | Categorical | None (0) / Primary (1) / Secondary or higher (2) |
| Husband/Partner’s education | Highest educational level attained by the respondent’s husband/partner | Categorical | None (0) / Primary (1) / Secondary or higher (2) |
| Husband/Partner’s age | Age of the respondent’s husband/ partner in years at interview | Continuous |  |
| Union | Type of union between the respondent and husband/partner | Categorial | Monogynous (0) / Polygynous (1) |
| ***Enabling*** | | | |
| Household wealth | A composite measure of a household's cumulative living standard, estimated by the survey using household's ownership of selected assets, such as televisions and bicycles; materials used for housing construction; and types of water access and sanitation facilities. It was grouped into five categories by DHS: Poorest, Poor, Middle, Rich and Richest. However, in this study it was re-categorized into: Poor (poor and poorest) / Middle / Rich (rich and richest) | Categorical | Poor (0) / Middle (1) / Rich (2) |
| Decision maker | Decision maker for using contraception | Categorical | Mainly respondent (0) / Joint decision (1) / Mainly husband or partner and Others (2) |
| Area of residence | Place of residence of the respondent at interview | Categorical | Urban (0) / Rural (1) |
| Media exposure | Hearing about family planning in the last few months from radio, or television, or newspapers or magazines | Categorical | Yes (0) / No (1) |
| ***Need*** | | | |
| Living children | Number of living children at interview | Continuous |  |
| Ideal and living children | Difference between number of living children and ideal number of children | Categorical | Living equal or greater than ideal (0) / Living less than ideal (1) |
| Number of sons | Number of living male children at interview | Continuous |  |
| Wantedness | Whether the last child born in the last five years was wanted at that time, later or not at all | Categorical | Wanted then (0) / Wanted later (1) / Wanted no more (2) |
| **Country level** | | | |
| ***Predisposing*** | | | |
| Literacy rate | The percentage of female population aged 15 years and over who cannot both read and write with understanding a short simple statement on his/her everyday life | Continuous |  |
| ***Enabling*** | | | |
| Births attended by skilled health providers | The percentage of deliveries attended by personnel trained to provide basic care to women and their newborns during [pregnancy](https://en.wikipedia.org/wiki/Pregnancy), [childbirth](https://en.wikipedia.org/wiki/Childbirth) and the [postpartum period](https://en.wikipedia.org/wiki/Postpartum_period) | Continuous |  |
| Density of medical doctors | Number of medical doctors per 10,000 population | Continuous |  |
| Rural population | Percentage of total population living in the rural area | Continuous |  |
| Gross national income | A measure of income of a nation’s residents and businesses, regardless of where it's earned (Atlas method) | Continuous |  |
| Poverty rate | Percentage of the population living on less than $1.90 a day at 2011 international prices | Continuous |  |
| Out-of-pocket expenditure | Percentage of total current health expenditure that is out-of-pocket payment | Continuous |  |
| ***Need*** |  |  |  |
| Total fertility rate | The number of children that would be born to a woman if she were to live to the end of her childbearing years and bear children in accordance with age-specific fertility rates of the specified year | Continuous |  |
| Under-five mortality rate | Number of deaths in children under five years of age per 1,000 live births | Continuous |  |
